# Characterizing Dielectric Permittivity of Nanoscale Dielectric Films by Electrostatic Micro-Probe Technology: Finite Element Simulations

**DOI:** 10.3390/s19245405

**Published:** 2019-12-07

**Authors:** He Ren, Wei-Feng Sun

**Affiliations:** Key Laboratory of Engineering Dielectrics and Its Application, Ministry of Education, School of Electrical and Electronic Engineering, Harbin University of Science and Technology, Harbin 150080, China; kingstel@163.com

**Keywords:** electrostatic probe, nanoscale dielectric film, dielectric permittivity, finite element simulation

## Abstract

Finite element simulations for detecting the dielectric permittivity of planar nanoscale dielectrics by electrostatic probe are performed to explore the microprobe technology of characterizing nanomaterials. The electrostatic force produced by the polarization of nanoscale dielectrics is analyzed by a capacitance gradient between the probe and nano-sample in an electrostatic detection system, in which sample thickness is varied in the range of 1 nm–10 μm, the width (diameter) encompasses from 100 nm to 10 μm, the tilt angle of probe alters between 0° and 20°, and the relative dielectric constant covers 2–1000 to represent a majority of dielectric materials. For dielectric thin films with infinite lateral dimension, the critical diameter is determined, not only by the geometric shape and tilt angle of detecting probe, but also by the thickness of the tested nanofilm. Meanwhile, for the thickness greater than 100 nm, the critical diameter is almost independent on the probe geometry while being primarily dominated by the thickness and dielectric permittivity of nanomaterials, which approximately complies a variation as exponential functions. For nanofilms with a plane size which can be regarded as infinite, a pertaining analytical formalism is established and verified for the film thickness in an ultrathin limit of 10–100 nm, with the probe axis being perpendicular and tilt to film plane, respectively. The present research suggests a general testing scheme for characterizing flat, nanoscale, dielectric materials on metal substrates by means of electrostatic microscopy, which can realize an accurate quantitative analysis of dielectric permittivity.

## 1. Introduction

At present, by means of a scanning microwave microscope (SEM), A nanoimpedance microscope, electrostatic microscope, scanning capacitance microscope and other microscopic techniques, the comprehension of material structure has reached the atomic scale [1,2,3,4]. The principle of these microscopic techniques have similar probe structures which characterize materials by scanning on the specimen surface with a needle tip to obtain information such as micromorphology, charge distribution, impurity state, dielectric permittivity, etc. [5]. It has become a key research field to quantify the dielectric permittivity of nanoscale objects by the scanning probe microscope. The quantitative analysis of intrinsic dielectric property can identify the material composition of nanoscale objects, which is essentially fatal in material science and biomedicine. 

However, measuring dielectric permittivity of nanoscale materials has always been a great challenge, particularly in the detection of an inherent weak dielectric signal with correlating the size, shape and non-local contribution region of the scanning probe [6].

Firstly in 2007, Fumagalli proposed a scheme for quantitatively detecting a local, low frequency dielectric constant of insulating thin films by a nanocapacitance microscope, the sub-micron spatial resolution of which is superior to standard technology, being promised to provide new insights for the physical and technical applications of dielectric materials on the nanometer scale [7]. Laura Fumagalli also suggested a technology called quantitative nanoscale dielectric microscopy in 2008, by which the low frequency dielectric constant of single-layer biomembranes can be noninvasively quantified and imaged with nanoscale spatial resolution, representing a great possibility of studying cell bioelectricity on a nanoscale [8]. Furthermore, the unmarked characterizations on the material composition of nanoparticles can be fulfilled by quantifying the inherent dielectric response to an applied electric field, as firstly reported also by Laura Fumagalli in 2012 [9]. The electrostatic microscope with sub-piconewton resolution can test the dielectric constant and internal composition of a single dielectric nanoparticle without any reference material, and can even explicitly distinguish the unmarked 10 nm nanoparticles with similar morphology but different low polarization materials, such as identifying the viral capsid containing DNA, making it possible to characterize nanoscale dielectrics and biological macromolecules in situ. Especially in 2018, Fumagalli reported local capacitance measurements of interfacial water confined between two atomically flat walls being separated by nanometers, and elucidated the anomalously low dielectric constant down to two, due to an interfacial layer with small vanishing polarization [10]. These results provide an excellent comprehension on surface dielectric interaction and demonstrated a strategic routine to study the dielectric properties of fluids and solids under extreme confinement. In addition, the dielectric constant of flagellin (diameter 10–20 nm) in flagellum hyphal can be measured by scanning dielectric microscope, which is suitable for characterizing nanoscale filamentous protein complexes and general 3D macro-molecular protein geometry, thus opening up an exploring way to correlate a dielectric response to protein structure and function [11].

For measuring the dielectric permittivity of nanoscale materials, excellent progresses have been achieved in scanning probe microscopy. The local dielectric spectra at a frequency range of 0.01–100 Hz deriving from the near-surface polarization of nanoscale materials have been tested by using a non-contact scanning probe microscope [12,13]. By exploiting the equivalent charge method and fitting experimental data, the local dielectric constant of nanoscale thin film has been quantitatively measured with an electrostatic force microscope (EFM), which can obtain the quantitative dielectric image of insulating nanofilm with a higher lateral resolution [14,15]. According to the electrostatic force from point charge or polarizable particles on the dielectric surface, Riedel suggested from numerical simulations that electrostatic force microscopy is capable of fulfilling the same lateral resolution for 100 nm dielectric film as for infinite dielectrics [16]. Mottaghizadeh analyzed the total capacitance between the probe tip and nanoscale dielectric material in EFM tests, by which the charge response of Fe_3_O_4_ nanoparticles was investigated [17]. Labardi proposed an extended model of the interaction between EFM probe tip and thin dielectric film on a conductive substrate, in which higher accurate dielectric permittivity was obtained by single-frequency measurements [18]. Taking the advantage of the high resonant frequency and low thermal noise of a subminiature cantilever beam, cantilever dynamics is correlated to the complex dielectric functions of nanoscale dielectrics at a frequency approaching to a few MHz [19]. In addition to extending the dielectric spectral range of atomic force microscopy (AFM), the method can also identify the electrostatic excitation frequency, thereby providing a higher dielectric contrast for characterizing nanomaterials.

The precise quantification of dielectric constant is mainly based on the theoretical modeling of electrostatic interaction between microprobe and tested nanosample, and is therefore substantially influenced by the geometry and size of the probe the and sample. It is not possible to find a general analytical formalism to solve this problem, due to the complexity of geometry and size, thus employing the numerical calculations encounters a great difficulty. 

Accordingly, a number of geometric models for simplifying the probe or sample are proposed to approximately analyze alternating current and the electrostatic force measured for characterizing the dielectric nanosample. At present, the state-of-art approaches of describing the electrostatic force between the atomic force microscopy probe and dielectric sample can be classified into Analytical Evaluation, Equivalent Charge Method and Finite Element Simulation. Based on a conductive tip capacitor system with high axis symmetry, Hudle proposed a theoretical analysis model to evaluate the electrostatic force on the top surface of probe tip in atomic force microscopy [20]. The Hudle model allows approximately quantitative analyses on the capacitance and electrostatic force of the tip surface, but cannot guarantee the high testing symmetry and needs to be improved in accuracy by numerical methods. According to the Equivalent Charge Method (ECM), Arinero investigated the nanoparticles filled into dielectric film composites by finite-element simulations, and demonstrated that the electrostatic force detected by a conductive probe can be quantitatively analyzed by ECM independent of film thickness, tip radius and tip-sample distance [21,22,23]. Boularas proved by the finite-element simulations of electrostatic force between AFM tip and dielectric surface that the tip geometry has a great influence on the electrostatic force detection [24]. By analyzing the truncated cone and cantilever of a microscopic probe in detecting the electrostatic interaction between the probe tip and a thick insulating substrate, Gramse presented an effective model for describing the electrostatic contribution from the cantilever [25]. Through a quantitative interpretation of the electrostatic force between a sharp silicon tip and the dielectric sample, Fumagalli suggested the double-angle-cone model in comparison to the traditional single-angle cone model, based on which the dielectric performances of nanoscale materials can be quantitatively analyzed with a higher resolution [26].

The most effective geometric model is the truncated cone containing all constitutes of the microprobe which can considerably promote an electrostatic interaction and accurately quantify the dielectric constant of the film sample with a thickness less than 1 μm or greater than 10 μm. In the modeling of planar dielectrics, the sample is usually constructed as laterally infinite to reduce the influence of plane size on the probe-induced electrostatic force. Although the accurately quantitative analysis cannot be carried out with the simplified model, the tested data from probe detection can be physically interpreted, which is qualified to represent the finite lateral dimension of dielectric nanofilm [27]. Under the action of electrostatic force, the electrostatic probe fluctuates up and down with the change of the sample surface profile and causes substantial vibrations of microcantilever during the EFM scanning process. Hence, the scanning EFM probe is not always perpendicular to the sample surface, leading to the asymmetric distribution of the electric field between the probe tip and sample. The two-dimensional axis-symmetric capacitance model is invalid in this case, and the electrostatic force detected by the tilted probe should be determined by the tilting angle. Therefore, it is necessary to carry out three-dimensional simulations of the actual probe operation in order to ensure the adequate accuracy of the electrostatic force measurement. The present research pertains to the influence of sample dimension and probe tilting on the electrostatic force sensed by the microprobe, which is studied employing finite-element simulations of the electric field in electrostatic probe microscopy.

## 2. Numerical Simulation and Analysis Scheme

### 2.1. Finite Element Simulation

**Figure 1:**
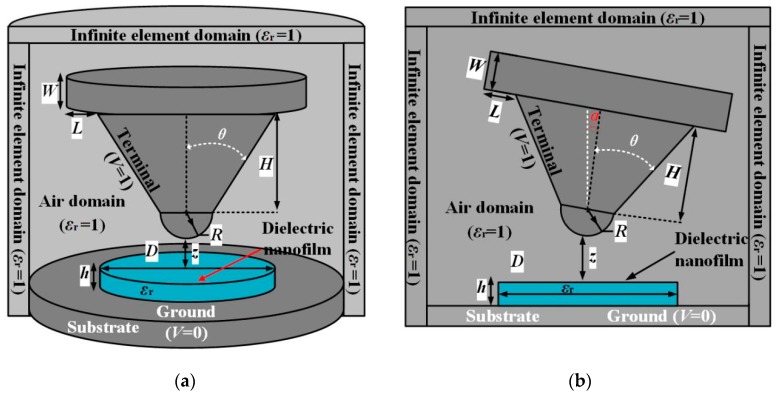
Schematic 3D model of probe detection system for electrostatic force microscopy characterizing nanoscale dielectric film: (**a**) the probe axis is perpendicular to film plane; (**b**) tilt of probe axis.

Three-dimensional electrostatic field simulations of planar nanoscale dielectrics detected by a microprobe detection system is carried out as implemented by electrostatic (AC/DC) module of COMSOL Multiphysics 5.4, investigating the influences of the lateral size of nanomaterial sample and the probe inclination on electrostatic force. As in the schematic diagram shown in Figure 1, the three-dimensional (3D) finite element model consists of a conductive microprobe in truncated cone geometry and a circular nanoscale dielectric film on metal substrate, in which the probe axis is perpendicular and tilting to the plane of the nanofilm sample, respectively. 

Beyond the metal-coated probe (30 nm in tip radius) and heavily-doped diamond probe (100 nm in tip radius), the heavily-doped silicon probe usually used in AFM can bear a sharp and stable tip in a radius of 1–10 nm, which has been recently proved capable of increasing the EFM lateral resolution in the scanning dielectric measurements of nanoscale materials [26]. Therefore, in our simulations, the microprobe is composed of a hemispheric tip with the radius *R* and a truncated cone with the height *H* and half-angle *θ*. The disk with radius equal to *L+H·*tan(*θ*) (*L* denotes the disc radial size that is not covered by the cone bottom) and thickness *W* at the probe base of a vertebral body is modeled to represent a cantilever. The probe tip is located at the distance of z away from above the surface of the planar disk sample with a diameter of *D* and a thickness of *h*. The inclination angle of the probe axis to the normal direction of the sample plane is identified by *α*.

The surface of probe tip is set to a voltage of 1 V as “terminal”, while the bottom of the metal substrate at the sample bottom is set as “ground” with zero voltage. The adequately large space around the probe is set as “air domain”, and the outermost environment is set as an infinite element domain that the top and side boundaries of the three-dimensional model space are set to be “zero charge” (**n**∙*E* = 0). Based on simulating electric potential distribution, electrostatic force can be evaluated by integrating the Maxwell-stress-tensor on the probe surface [9]. The specific microscopic parameters adopted for the probe are as follows: truncated cone height *H* = 12.5 μm, cantilever radius *L* = 1 μm, cantilever thickness *W* = 2 μm. Three kinds of probes with different geometry are simulated: (1) *R* = 100 nm and *θ* = 30°; (2) *R* = 100 nm and *θ* = 25°; (3) *R* = 10 nm and *θ* = 25°. The thickness and width (diameter) of the investigated dielectric nanofilm both vary from 1 nm to 10 μm and from 100 nm to 10 μm, respectively, with their relative dielectric constant being altered between 2 and 1000 to completely cover all kinds of dielectric materials.

In the finite element simulations of EFM testing system using COMSOL, the free triangular mesh generation is employed to refine local meshes at the positions where the electric field greatly changes. According to the Delaunay triangulation algorithm, the model is divided into 880,271 elements in total with the maximum and minimum elements being modified until the obtuse-angle triangulation has been eliminated. The element growth rate is set to 1.5, and the relaxation degree of the narrow region is set to 1 in mesh generation. The adopted mesh generation allows us to achieve finite-element solutions independent of element size.

### 2.2. Electrostatic Force Analysis Scheme

An electrostatic microprobe is a scanning probe microscope which uses the electrostatic interaction between the sample and a conductive metal probe to characterize charge distribution, surface potential and relative dielectric constant. The electrostatic microscope operates with the similar principle as the atomic force microscope, which is mainly composed of a cantilever beam and probe, while sensing and imaging by measuring the electrostatic interaction between the probe and sample that is different from the van der Waals force as the atomic force microscope. When an operating voltage is applied between the surfaces of the conductive probe and metal substrate so as to generate electrostatic interaction between the probe and sample, the cantilever probe is oscillating on a sample surface under electrostatic force without directly contacting with sample as the sample being scanned [28]. Assuming that the potential difference between the metal probe and sample is *V*, and the equivalent capacitance is *C*, the total electrostatic energy of this detecting system can be formalized as:

(1) $$W=\frac{1}{2}CV^{2},$$

where *z* denotes normal distance from the sample surface. When *z* is varied under a constant potential difference *V*, the equivalent capacitance *C* will accordingly change, resulting in a change of electrostatic potential energy which is equivalent to the work done by electrostatic force on the probe displacement. Thus the electrostatic force on this probe is derived as:

(2) $$F_{z}(z,\varepsilon_{r})=\frac{\partial W}{\partial z}=\frac{1}{2}\frac{\partial C}{\partial z}V^{2},$$

According to the formula of electrostatic force, the capacitance gradient is obtained by *C’=2F_z_(z,ε*_r_*)/V*^2^ (*ε*_r_ symbolizes the relative dielectric permittivity of the tested nanomaterial), which is preferable rather than electrostatic force in the advantage of providing all of the information about electrostatic interactions and being independent of the applied potentials [29]. Therefore, in order to improve the measurement accuracy by reducing the electrostatic contributions uncorrelated with the local dielectric polarization of the tested nanosample, we employ relative the capacitance gradient *ΔC’(z,ε*_r_*)=C’(z,ε*_r_*)-C’(z*_ref_*,ε*_r_*)* as a function of the characterizing dielectric constant to represent differential capacitance caused by probe normal displacement along the *z* direction from the reference tip position *z*_ref_, which denotes the reference distance of the probe tip with respect to the nanofilm sample (usually *z*_ref_ = 100 nm) [30].

## 3. Results and Discussion

### 3.1. Film Lateral Dimension and Probe Tilt Angle

**Figure 2:**
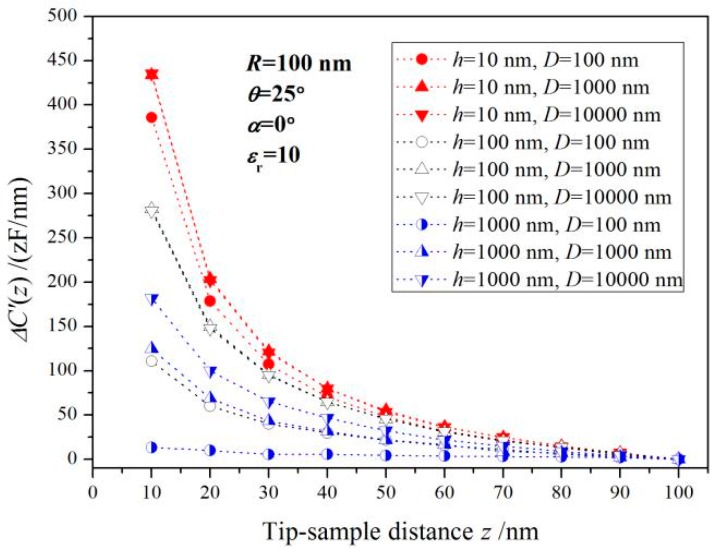
Capacitance gradient varying with the distance between probe and sample.

**Figure 3:**
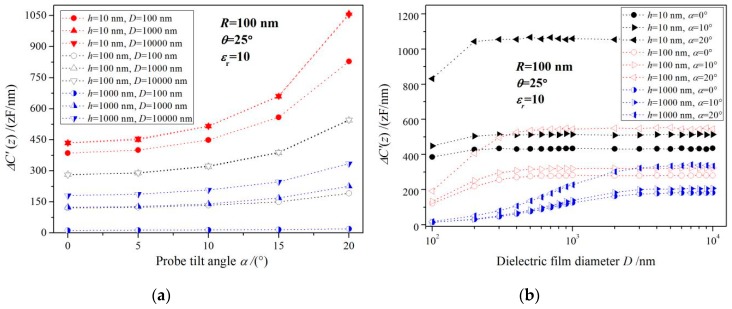
Capacitance gradient curves as a function of (**a**) probe tilt angle and (**b**) dielectric nanofilm thickness.

Based on the simulated capacitance of the probe detecting system and Equations (1) and (2), capacitance gradients measured by the probe are calculated as a function of the tip dielectric distance for three kinds of nanofilm samples with different thickness and plane dimension, as the curves shown in Figure 2, which are varying flatly, and gradually converge to a constant value at long distances from the metal substrate. For a given film thickness, *ΔC’* increases with the enlargement of the sample lateral size (nanofilm diameter), and becomes unchanged when this nanofilm diameter exceeding the critical diameter *D*c which represents the minimum diameter that the tested nanofilm can approximately be considered as infinite in lateral dimension when the dielectric signal *ΔC’* increases to 99% of the maximum convergence value with the sample diameter being raised [30]. It is thus concluded that *ΔC’* is independent of the nanofilm diameter when it exceeds the critical diameter. By the inclining electrostatic probe, it is found that the measured *ΔC’* increases with the tilting angle of probe axis until it converges to a stable value, as shown in Figure 3a exhibiting the capacitance gradient varying as a function of probe tilt angle for dielectric film in thicknesses of 10, 100 and 1000 nm. 

Furthermore, similar saturation features in curves of *ΔC’* vs. sample lateral dimension are also represented for the tilting probe as shown in Figure 3b, indicating that the capacitance gradient remains constant and independent of the nanofilm diameter when the sample lateral size is larger than *D*c. It is eventually noted that the thinner nanofilm sample will suffer more sensitively from the influence of the probe tilting angle on *ΔC’*.

**Figure 4:**
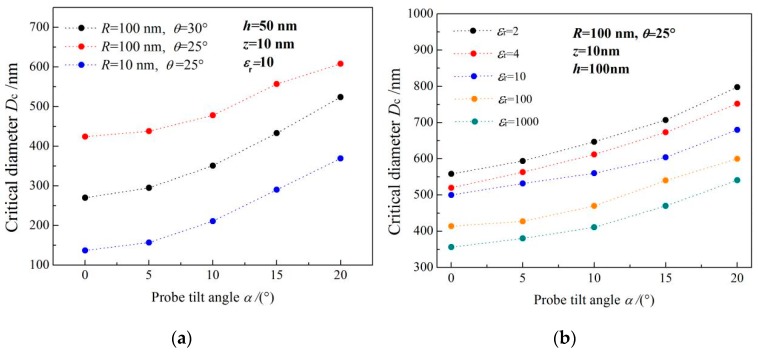
Critical diameter of dielectric nanofilm vs. probe titling angle for (**a**) three probe tip geometries and (**b**) different dielectric constants of sample.

*D*c is not only the function of the thin film thickness, but also a function of the probe tilting angle, as the relationship between the tilting angle of the electrostatic probe and the critical diameter of dielectric nanofilm shown in Figure 4 for three probe tip geometries (a) and different dielectric constants of sample (b). The critical diameter of nanofilms (*h* < 100 nm) is greatly affected by the size of the probe tip, so we adopt probe tips with discrepant radius in the simulations of electrostatic force detection for *h* = 50 nm thin film. Figure 4a shows that the probe tilt also has a significant influence on *D*c due to the almost zero electric potential of the whole nanofilm which is very close to conductive substrate on both sides. The lateral dimensions of the dielectric sample for accurate EFM measurement are required to be larger than a critical value in all the directions perpendicular to the substrate normal. Therefore, probe tilting will cause an inevitable reduction of *D*c for circular film, due to the most increased critical lateral size along the probe tilting direction where the highest electric field with a largest potential gradient is distributed. However, the critical diameter of a thicker dielectric film (*h* > 100 nm) is evidently affected by dielectric permittivity. As illustrated by Figure 4b, the critical diameter of *h* = 100 nm films with different dielectric constants increases with probe tilting angle. 

The probe tilting will affect the critical diameter for all the dielectric samples from ultra-thin film (*h* < 10 nm) to macroscopic film. Both probe tip geometry and probe axis inclination are fundamental to determine the critical lateral dimension of the sample in the nanoscale dielectric measurements. The probe tilt also significantly affects the detected dielectric signal in EFM tests, as shown in Figure 3. The dielectric signal almost increases exponentially with the increase of the probe tilt angle, especially the ultrathin film, which can also be attributed to the *D*c increment caused by probe tilting.

**Figure 5:**
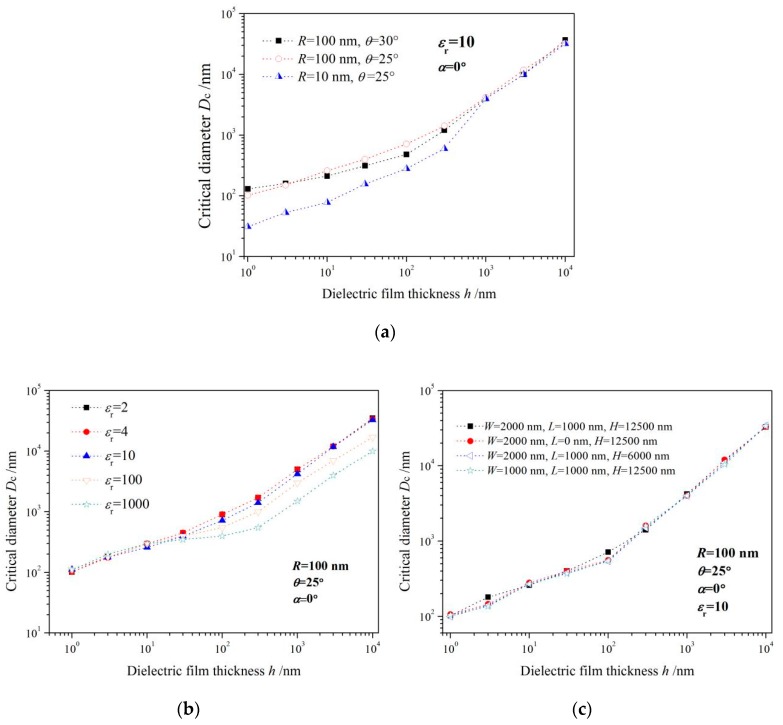
Critical diameter varying with specimen thickness when altering (**a**) probe tip geometry, (**b**) relative dielectric constant of specimen, (**c**) cantilever beam dimension.

**Figure 6:**
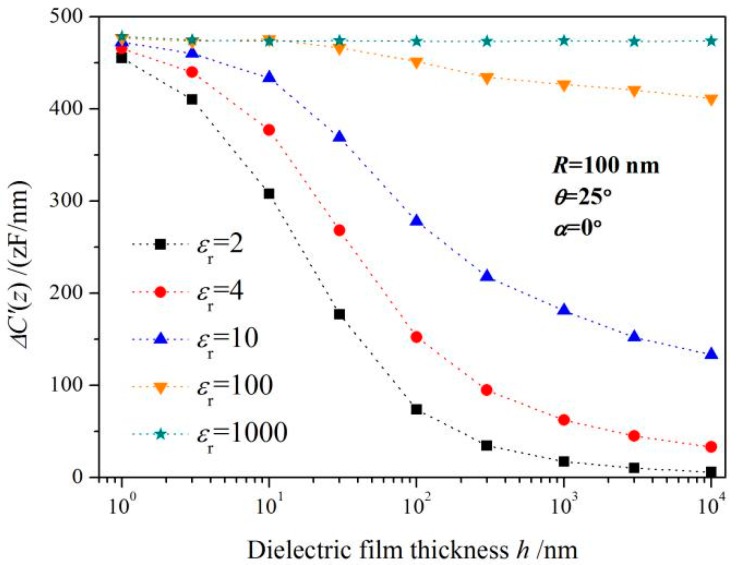
Variation curve of capacitance gradient vs. specimen thickness for different relative dielectric permittivity of nanofilms.

As the critical diameter dependence on film thickness for three representative probe geometries shown in Figure 5a, the influence of the tip radius *R* and cone angle *θ* on the critical diameter can be generally evaluated by these three geometric paradigms. *D*c should be considered as an increasing function of film thickness in theoretical modeling, as it may lead to systematic errors in the analysis of experimental data. Besides, *D*c also depends on the nanoscale geometry of the expected probe, but only in a limited film thickness range (typically for *h* < 1 μm). While for thicker films (*h* > 1 μm), the *D*c is almost independent of the microprobe geometry as *R* and *θ*, which does not mean that the electrostatic force itself is independent of the microprobe geometry. The electrostatic force is usually dependent upon *R* and *θ*, but almost unaffected by probe geometry when the lateral size of this nanosample can be regarded as infinite. Figure 5b provides the critical diameter dependence on the dielectric permittivity of nanomaterials for a fixed probe geometry (*R* = 100 nm, *θ* = 25°). It is explicitly indicated that the overall altering trend of *D*c with the thickness of nanofilm is very similar for various dielectric constants. For film thickness less than 10 nm, *D*c is almost not affected by the dielectric permittivity of the nanosample, while for thicker films, *D*c lies more likely on the dielectric permittivity. We have also investigated the geometrical effects deriving from the cantilever size and cone height of the microprobe on *D*c, which implies the cantilever size and cone height had no significant impact on *D*c within a comprehensive range of film thicknesses, as shown in Figure 5c. By simulating the dielectric signal *ΔC’* of microprobe detecting planar nanomaterials when its lateral dimension in raised larger than *D*c, it is found that the electrostatic forces are evidently rested with the thickness and dielectric permittivity of those nanosamples, particularly for a higher dielectric constant, as shown in Figure 6.

### 3.2. Electric Potential Distribution Analysis

**Figure 7:**
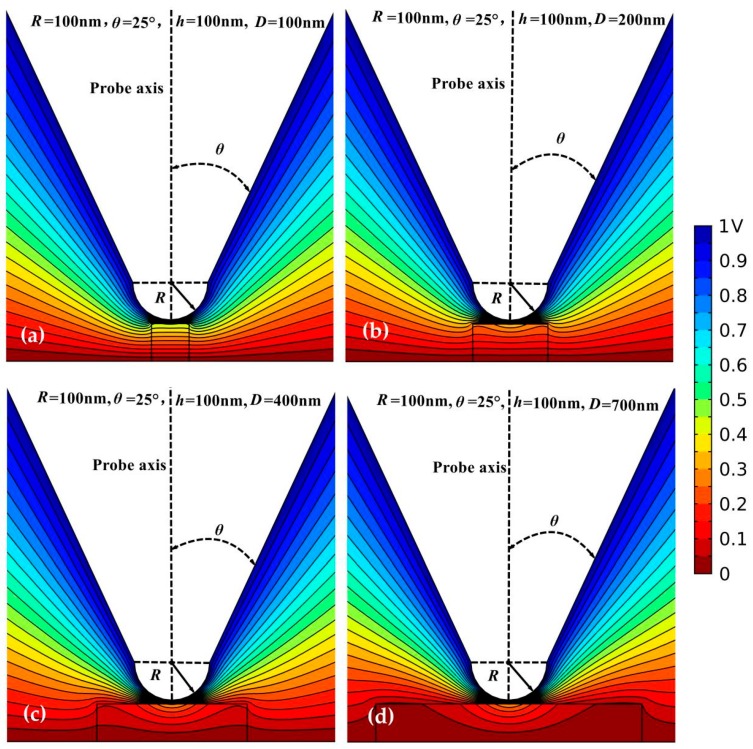
Electrostatic potential distributions in EFM for perpendicular probe with a geometry of *R* = 100 nm and *θ* = 25°, and the dielectric nanofilm with a thickness of *h* = 100 nm and a relative dielectric constant of *ε*_r_ = 10, in different diameters of (**a**) 100 nm; (**b**) 200 nm; (**c**) 400 nm; (**d**) 700 nm.

The underlying physical mechanism in the process of probe detection can be represented by analyzing the electric field as shown in Figure 7, illustrating the simulated spatial distribution of electrostatic potential between the microprobe (*R* = 100 nm; *θ* = 25°) and dielectric nanofilm (*h* = 100 nm; *ε*_r_ = 10; *D* = 100, 200, 400, 700 nm) in electrostatic force microscopy (EFM). 

It is indicated that the electrostatic potential distribution below the apex of the probe obviously depends on the lateral size and thickness of the tested nanomaterial. For nanofilms with small diameters, a significantly decreasing potential distributes in the whole nanoscale dielectrics; while for large diameters, the electric potential drop is more likely concentrated in the location directly below the tip vertex. The potential distribution in nanoscale dielectrics not only originates from the voltage applied between the probe tip and top surface of metal substrate, but also depends on the potential variation across the side surfaces of nanomaterial. When the lateral dimension (film diameter) of the nanomaterial is constrained in a very small range so as to be comparable to its longitudinal dimension (thickness), a majority of side surfaces is far away from the bottom electrode where the potential approximates to zero, and is relatively close to probe tip where the potential is substantially higher than zero, resulting in a nonzero potential boundary condition across the side surface of the tested nanomaterial and a significant potential distribution inside it. On the contrary, when the lateral size of nanoscale dielectrics is greatly larger than its longitudinal thickness, the side surfaces far away from the probe tip are completely encompassed into zero potential domain in consistence with a zero potential boundary condition, leading to a diameter-independent potential drop crossing nanomaterials. Therefore, the potential drop inside nanomaterials will not further increase when its lateral size is raised to a critical value so that the potential on side surfaces approaches to zero, which accounts for the existence of *D*c for characterizing nanoscale dielectrics. Furthermore, with the decreases in film thickness, probe cone angle and tip radius, it is more difficult for side surfaces to reach the zero potential boundary condition, implying that *D*c can be reduced by decreasing the longitudinal dimension of the nanoscale dielectrics or refining probe geometry.

**Figure 8:**
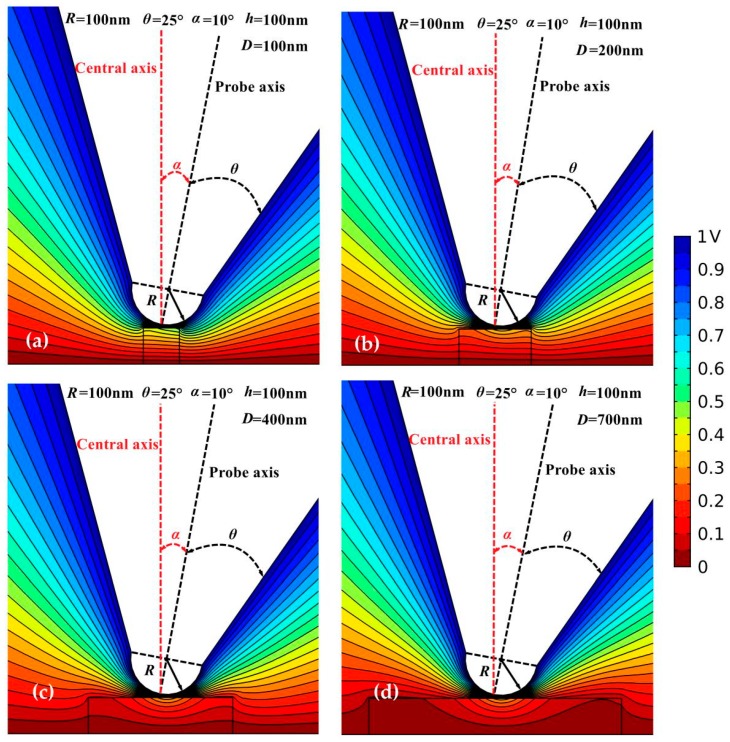
Electrostatic potential distributions in EFM for tilting probe with a geometry of *R* = 100 nm and *θ* = 25°, and the dielectric nanofilm with a thickness of *h* = 100 nm and a relative dielectric constant of *ε*_r_ = 10, in different diameters of (**a**) 100 nm; (**b**) 200 nm; (**c**) 400 nm; (**d**) 700 nm.

When the axis of the electrostatic probe is slightly tilting from correct verticality, the nanosamples will be polarized by the non-uniform electric field in a non-central symmetry, which is explicitly illustrated from the electric potential lines, as shown in Figure 8. It is noted that the contour lines of electric potential are always denser on the side of the probe tilting than the counter side. Hence, when one side of the nanoscale dielectric film reaches zero potential, there is still a nonzero potential on the other side surface, which means a larger *D*c than that for vertical probe axis is required to simultaneously satisfy the zero-potential boundary conditions on both sides. This result clearly explains the influence of probe inclination on *D*c of nanoscale dielectrics in electrostatic probe microscopy. On the other hand, except under the condition that polarization of dielectric nanomaterial is directly affected by the probe tip (minimal radius), for the dielectric films with a nanoscale thickness, the side surface is very close to the bottom electrode of zero potential. Therefore, when the side surfaces of the dielectric film extend beyond the tip area, the side potential approaches to zero, leading to a total potential drop across the nanomaterial independent of lateral dimension. Thus, the nanoscale geometry of the microprobe is a dominant factor restricting the lower limit in lateral size of nanoscale dielectrics that can be accurately characterized by electrostatic probe microscopy. In complement, the side potential of the nanofilm does not approach to zero due to electrostatic interactions when the electrostatic probe is tilting, leading to a higher *ΔC’* detected by probe for the nanofilm with a smaller thickness, which consistently accounts for the results shown in Figure 3b.

### 3.3. Establishment and Verification of Analytical Equations

At the limiting condition of characterizing nanofilms that the polarization forces are only dependent of the probe geometry in nanoscales, a reasonable analytical expression can be deduced for electrostatic force. The capacitance gradient detected in EFM only encompasses three dominant contributions to the electrostatic force signal from probe apex (tip), cone and cantilever [4,7]:

(3) $$C_{\mathrm{EFM}}^{\prime }(z,\varepsilon_{r})=C_{\mathrm{apex}}^{\prime }+C_{\mathrm{cone}}^{\prime }+C_{\mathrm{cantilever}}^{\prime }$$

in which the probe nanodisplacement contributed from the cantilever can be ignored in comparison to cone and tip [25]. However, this is not the case for the cone bottom, particularly when the conical contributions play an important role for tips with relatively small radius (*R* < 50 nm) and small distance between tip and nanosample (*z* < 100 nm) [20]. It is also found that the probe tilt substantially influences on the critical diameter of the dielectric nanofilm and the detected electrostatic force. Simulation results verify that probe tilt has a notable influence on *D*c of the dielectric film due to its great impacts on the detected electrostatic force. Therefore, in order to accurately interpret EFM data, the analytical model representing the change of capacitance gradient should include the contributions from the probe tip and truncated cone, as well as the error caused by probe tilting, which can be represented as the following:

(4) $$\Delta C_{\mathrm{EFM}}^{\prime }(z,\varepsilon_{r})=\Delta C_{\mathrm{apex}}^{\prime }(z,\varepsilon_{r})+\Delta C_{\mathrm{cone}}^{\prime }(z,\varepsilon_{r})+\Delta C_{\mathrm{cantilever}}^{\prime }(z,\varepsilon_{r})+\Delta C_{\mathrm{derivation}}^{\prime }(\alpha ,z,\varepsilon_{r})$$

based on which a new analytical formula is here suggested for describing tip-sample capacitance for detecting dielectric nanofilms [4]:

(5) $$C_{\mathrm{tip}}(z,\varepsilon_{r})=2\pi \varepsilon_{0}R\cdot \ln[1+\frac{R\cdot (1-\sin\theta )}{z+h/2\varepsilon_{r}}]+c_{0}$$

where *ε*_0_ denotes dielectric permittivity of free space, and *c*_0_ is a correction constant. By calculating the derivative of the Equation (5), the capacitance gradient of probe apex can be obtained as following:

(6) $$C_{\mathrm{tip}}^{\prime }(z,\varepsilon_{r})=\frac{2\pi \varepsilon_{0}R^{2}\cdot (1-\sin\theta )}{\left(z+h/2\varepsilon_{r})(z+h/2\varepsilon_{r}+R\cdot \sin\theta \right)}$$

for the contribution from the probe cone, the electrostatic formula for metal substrate is extended to dielectric thin film by replacing z with *z+h/ε*_r_ [30], so that the contribution from the probe cone can be formulized as:

(7) $$C_{\mathrm{cone}}^{\prime }(z,\varepsilon_{r})=\frac{2\pi \varepsilon_{0}}{{\left[\ln\tan(\theta /2)\right]}^{2}}\{\ln[\frac{H}{z+h/2\varepsilon_{r}+R\cdot (1-\mathrm{Sin}\theta )}]-1+\frac{R\cdot \cos^{2}\theta /\sin\theta }{z+h/2\varepsilon_{r}+R\cdot (1-\mathrm{Sin}\theta )}\}$$

(8) $$C_{\mathrm{deviation}}^{\prime }(\alpha ,z,\varepsilon_{r})=2\pi \varepsilon_{0}\cdot \exp[\frac{\pi R\cdot \tan\alpha }{z+h/2\varepsilon_{r}+R\cdot (1-\sin\theta )}]$$

in which all the parameters have been determined. For large tip and the small distance between tip and metal substrate (*R* > 100 nm, *z+h/2ε*_r_ < 100 nm), the cone contribution in Equation (7) is almost independent of *z* so that its variation with probe displacement can be ignored. Therefore, it is not necessary to include cone contribution so that the tip contribution given by Equation (6) can be accurately used to determine the approximate test curves [31]. However, when using a smaller tip (*R* = 30–50 nm), both the apex and cone structures must be considered. 

**Figure 9:**
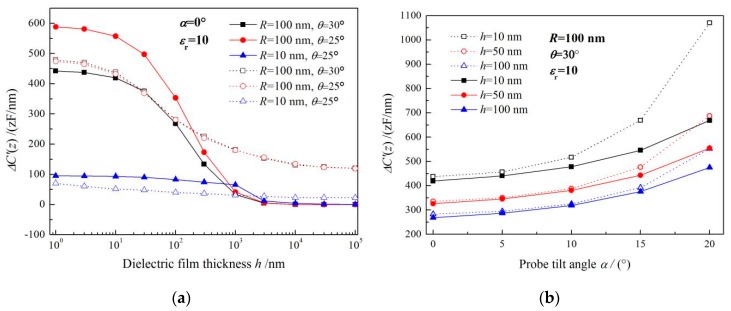
Capacitance gradient calculated by fitting analytical equation as a function of specimen thickness for (**a**) perpendicular and (**b**) tilting probes.

Although employing *z+h/2ε*_r_ instead of *z* is accurate for parallel plate geometry, it is not necessary valid for other geometry, so the effective range of exploiting Equations (6–7) must be evaluated [32,33]. Comparing the results calculated by the analytical Equations (6–7) (solid symbols) and the results of finite element numerical simulations (hollow symbols) as shown in Figure 9a, it is suggested for the ultrathin film (*h* = 10–100 nm) and appropriate probe-tip geometry (*R* = 100 nm, *θ* = 30°) without perpendicular probe-axis (*α* = 0°) that the analytical solutions agree well with the numerical simulations. In particular for influence of probe inclination on detecting the dielectric permittivity of planar nanomaterials by EFM, the error correction for the analytical formula is proposed in this paper. Based on the exponential and inverse relationship of the probe tilting angle *α* and nanofilm thickness *h*, respectively, with the tested dielectric signal, as shown in Figure 4, we propose the tilt-correction item with the similar logarithmic formalism as Equations (6–7) for the capacitance gradient error caused by probe tilting, as presented by the Equations (4) and (8) describing the detected dielectric signal as a function of probe tilting angle *α*. It is noted from Figure 9b that the curves calculated by the analytical formula with tilt-error correction are in good agreement with the curves obtained by finite-element simulations at small tilt angles (*α* < 10°), especially for thicker samples (*h* = 50,100 nm). The generalized analytical formula expressed by the Equations (4–8) can be used to quantitatively predict the electrostatic force between the metal probe and the dielectric nanofilms, where the lateral dimension *D* is larger than a critical value *D*c and its thickness is in the range of *h* = 10–100 nm (determined by *D*c), and the tilt angle of probe is less than 10°. When the probe detection condition is not qualified for these constrains, the analytical expression cannot be used to accurately calculate the capacitance gradient, which needs to be further improved by numerical simulations.

## 4. Conclusions

Finite element numerical simulations of a three-dimensional electrostatic field are performed to evaluate the Maxwell stress tensor on the probe surface as implemented by electrostatic (AC/DC) module of COMSOL Multiphysics 5.4 software package, in which the electrostatic force between the conductive probe and the flat nanoscale dielectric film with finite lateral size is systematically analyzed. For a given nanoscale dielectric film, the critical diameter depends, not only on the geometric structure and tilting angle of detecting probe, but also on film thickness. Especially for the dielectric films with thickness greater than 100 nm, the critical diameter is almost independent of probe geometry while being primarily determined by the thickness and dielectric constant of the tested film specimen, which follows the varying trend of an exponential function. Our simulations present a quantitative analytical formula for testing the dielectric permittivity of nanofilm by electrostatic force microscopy, which can be employed for in thin films with the thicknesses of 10–100 nm when the probe axis is restricted in a small inclination angle less than 10°. The analytical formalism is valid for a large tip (radius > 100 nm and cone angle = 30°) in a well consistence with finite-element simulations, which can be accurately utilized for quantitatively estimating the dielectric permittivity of the nanofilm in electrostatic force measurements. Whereas, the thicker materials with a thickness >100 nm lack a suitable quantitative analysis formula for describing electrostatic interactions. Therefore, it is necessary to exploit numerical simulations of precisely controlled specimen dimension and probe geometry to provide the theoretical basis and improvement schemes for the electrostatic probe technology used in dielectric characterizations of nanoscale films.
